# Label-free single-cell protein quantification using a drop-based mix-and-read system

**DOI:** 10.1038/srep12756

**Published:** 2015-08-03

**Authors:** Alireza Abbaspourrad, Huidan Zhang, Ye Tao, Naiwen Cui, Haruichi Asahara, Ying Zhou, Dongxian Yue, Stephan A. Koehler, Lloyd W. Ung, John Heyman, Yukun Ren, Roy Ziblat, Shaorong Chong, David A. Weitz

**Affiliations:** 1 School of Engineering and Applied Sciences, Harvard University, Cambridge, MA 02138, USA; 2 Department of Cell Biology, Key Laboratory of Cell Biology, Ministry of Public Health, and Key Laboratory of Medical Cell Biology, Ministry of Education, China Medical University, Shenyang 110001, China; 3 School of Mechatronics Engineering, Harbin Institute of Technology, Harbin 150001, China; 4 Department of Physics, Harvard University, Cambridge, MA 02138, USA; 5 New England Biolabs, Inc. 240 County Road, Ipswich, MA 01938, USA

## Abstract

Quantitative protein analysis of single cells is rarely achieved due to technical difficulties of detecting minute amounts of proteins present in one cell. We develop a mix-and-read assay for drop-based label-free protein analysis of single cells. This high-throughput method quantifies absolute, rather than relative, amounts of proteins and does not involve antibody labeling or mass spectrometry.

The average abundance of proteins in a single mammalian cell is estimated to be 100,000 copies^1^, corresponding to 16 femtomolar if the cell is lysed in a typical 10 microliter microwell assay. Conventional quantitative immunoassays have picomolar detection limits at best^2^, and therefore, lack the sensitivity to quantify proteins at the level of single cells. However, drop-based microfluidics enable single-cell encapsulation in picoliter water-in-oil drops^3^. Accordingly, the in-drop concentration of a mammalian protein with average abundance in a single cell exceeds 1 nanomolar, which is well within the detection range of conventional quantitative immunoassays. However, a general method suitable for determining protein concentrations in single drops has yet to be developed^3^.

The conventional method for single-cell protein analysis is fluorescence-based flow cytometry^4^, which often uses highly specific antibodies to label protein targets in single cells. Though the absolute amounts of proteins from one cell are extremely small, the local concentrations are sufficiently high to allow proteins to be detected as long as cells remain intact and proteins are specifically bound by fluorescent antibodies. This is normally achieved by cell fixation, permeablization, labeling and washing prior to the cytometric analysis. Currently up to 10–18 different protein species can be measured from single cells by multi-color flow cytometers^5^. To further increase the number of measurable parameters, antibodies conjugated with metal isotopes of different masses are used in mass cytometry^6^, which combines flow cytometry with mass spectrometry, allowing simultaneous profiling of 20–30 protein species per cells. In spite of these recent advances, cytometric technologies provide only a snapshot of cellular information at a given time and often cannot effectively measure secreted proteins^7^. Microencapsulation technologies, such as drop-based microfluidics^8^,^9^ and dense arrays of subnanoliter wells^10^, have the advantages of analyzing live single cells confined in picoliter volumes, thus allowing measurements over a period of time. Picoliter microfluidic drops can encapsulate both single cells and their secreted products. These secreted products are captured by antibodies immobilized on co-encapsulated beads or agarose gel^8^,^9^. Alternatively, single cells are trapped in arrays of subnanoliter wells and secreted products are captured and detected by antibodies on glass slides^10^. Similar to cytometric technologies, these microencapsulation technologies also require highly specific antibodies for both capturing and labeling targets.

Here we introduce a Protein Assay via Induced Gene Expression (PAIGE), which is a one-pot mix-and-read assay that enables quantitative protein measurements of single cells encapsulated in drops. Unlike all other single cell protein analysis methods, PAIGE does not require antibodies to label and capture target proteins from single cells, thus eliminating the steps of fixing, labeling and washing cells or immobilizing capturing antibodies. Another distinct feature of PAIGE is its ability to quantify absolute, rather than relative, amounts of proteins from single cells.

## Results and Discussion

**Figure 1:**
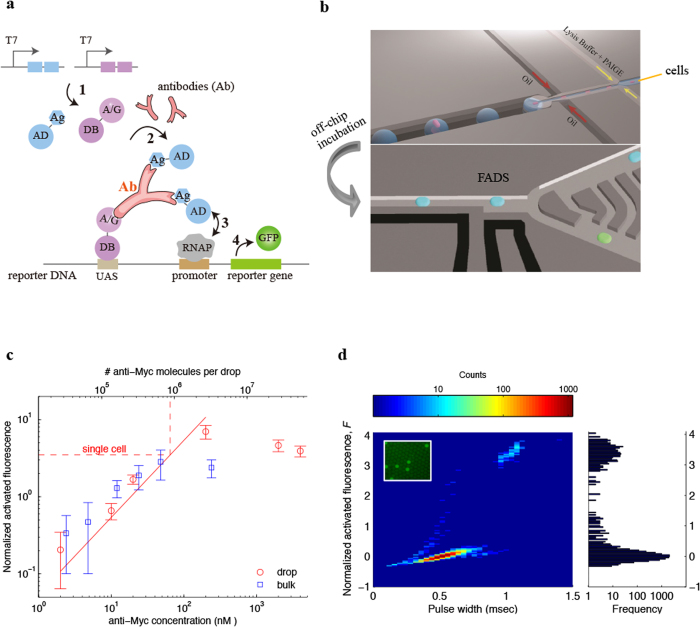
Detection and quantification of antibody in single hybridoma cells using drop-based PAIGE. (**a**) Scheme of PAIGE for detecting an antigen (Ag)-specific antibody (Ab). Two fusion proteins, AD-Ag and A/G-DB, are constitutively expressed under T7 promoter (T7) from input DNA templates by T7 RNA polymerase (step 1). The antibody binds to both Ag and A/G, forming a ternary complex that binds to the upstream-activation sequence (UAS) on the reporter DNA and recruits AD near the promoter-bound RNA polymerase (RNAP) (step 2). AD activates RNAP (step 3) to express the reporter gene, which produces GFP (step 4). (**b**) The workflow of drop-based PAIGE. Single cells (megenta) are encapsulated with PAIGE and a lysis buffer in drops (blue). After off-chip incubation, drops are re-injected into a fluorescence-activated drop-sorting (FADS) device to measure their fluorescence and sort them based on a fluorescence threshold. (**c**) Titration curves of pure anti-myc in microwell (blue square) and drop-based (red circle) PAIGE. The dashed line shows the average number of anti-Myc molecules in a single cell. The fluorescence of anti-Myc in microwells or drops is normalized by that of microwells or drops without anti-Myc. The normalized activated fluorescence, *F*, is obtained by subtracting one. (**d**) Heat map showing the distribution of drops in terms of their normalized activated fluorescence, *F*, and width recorded as the PMT’s pulse duration (left). The corresponding fluorescence histogram is shown on the right. The inset fluorescence microscopy image shows that after incubation a small fraction of the drops is bright.

PAIGE is derived from a recently developed IVT2H system that couples interactions between fusion proteins expressed from DNA templates with the activation of a fluorescent reporter gene^11^. To demonstrate PAIGE, we customize it for the detection of a target antibody specific to an *in vitro* expressed epitope (Fig. 1a). The target antibody (Ab) forms a ternary complex bridging two *in vitro* expressed fusion proteins. The first fusion protein (AD-Ag) contains the activation domain (AD) fused to the antibody epitope (Ag). The second fusion protein (A/G-DB) contains the hybrid protein A/G fused to the DNA binding domain (DB). The resulting antibody ternary complex binds to the promoter region of the reporter DNA and activates expression of the fluorescent GFP reporter (Fig. 1a). We use monoclonal anti-Myc, which specifically binds the c-myc epitope, as a model system. In microwell assays, PAIGE expressing c-myc epitope in the fusion protein AD-myc activates the GFP expression only in the presence of anti-Myc, but not different antibodies, such as anti-MBP (Supplementary Fig. 1). In the absence of anti-Myc, the two expressed fusion proteins (AD-myc and A/G-DB) do not interact and consequently only a basal level of GFP is expressed (Supplementary Fig. 1). Furthermore, the fluorescence of PAIGE in microwell assays is linearly correlated to the concentration of anti-Myc (Fig. 1c, blue square, bulk). These data establish that PAIGE expressing a given epitope can be used to detect and quantify an antibody that is specific to the epitope.

We apply PAIGE in drop-based microfluidics, whose workflow includes encapsulation, off-chip incubation, fluorescence detection and drop sorting (Fig. 1b). We first encapsulate PAIGE with different concentrations of pure anti-Myc in drops and measure their fluorescence after incubation. We observe a linear correlation of drop fluorescence with anti-Myc concentrations ranging from 2 nM to 200 nM, corresponding to 30,000 to 2,000,000 anti-Myc molecules per drop (Fig. 1c, red circle, drop). Remarkably, this linear correlation is in good agreement with that of bulk measurements in microwells, in spite of ~500,000 fold reduction in volume. In both drop and bulk experiments, we observe large errors (standard deviations) at ~2 nM of anti-Myc (or 30,000 molecules per drop), suggesting that the sensitivity of PAIGE for anti-Myc is at the low nM range. Such sensitivity is comparable to that of the conventional ELISA in microwells. However, by performing in drops, PAIGE has the sensitivity of detecting proteins from single cells. We also observe a saturation or slight decrease in the fluorescence of PAIGE at very high concentrations of anti-Myc (e.g., >2,000,000 molecules per drop), which we attribute to a large number of anti-Myc binding to both fusion proteins that prevents further formation of antibody ternary complexes (Fig. 1a). However, it is estimated that most mammalian proteins have less than 2,000,000 copies per cell^12^.

To demonstrate single-cell antibody quantification, we co-encapsulate 9E10 hybridoma cells that produce anti-Myc, with PAIGE and a cell-lysis agent in drops (Fig. 1b). We dilute these cells to achieve a Poisson loading such that 10% of drops contain single cells, 90% are empty, and a negligible number of drops contain multiple cells. After incubation, the heatmap of the fluorescence from 10,000 drops exhibits a bimodal distribution in which ~10% of drops are bright (Fig. 1d). We sort individual bright drops and verify that they contain 9E10 cells by RT-PCR amplification of the β2-microglobulin gene, which is expressed constitutively in all hybridoma cells, followed by Sanger sequencing (Supplementary Fig. 2). Based on the linear correlation of drop fluorescence with anti-Myc concentrations, we determine that bright drops contain on average 880,000 anti-Myc molecules in a single hybridoma cell (Fig. 1c, dash line). The contents from lysed cells could affect the PAIGE signal in drops, and the accuracy of the estimate that is based on the linear correlation of pure anti-Myc. However, the cell lysate is diluted ~8-fold in drops in the PAIGE assay. Moreover, we believe that the mammalian factors are unlikely to significantly affect the bacterial transcription and translation machineries in PAIGE. Additionally, our result agrees with an ensemble estimate of the amount of anti-Myc secreted from a bulk 9E10 culture, which is on average 1,000,000 anti-Myc per cell (John Heyman, unpublished). To the best of our knowledge, this is the first drop-based single-cell quantification of an antibody, and the first high-throughput single-cell quantification of a protein that is not a fluorescent protein or an enzyme whose product is a fluorescent molecule^13^. By further optimizing PAIGE, such as increasing the binding affinity towards the target molecule, we may improve its sensitivity at the low nM range, which corresponds to ~30,000 molecules per cell. Since the average abundance of proteins in a single mammalian cell is estimated to be 100,000 copies^1^, drop-based PAIGE can potentially be used to measure medium to low abundance proteins from single cells.

In principle, drop-based PAIGE can be customized to detect and potentially quantify any molecule of interest from single cells, provided that appropriate binders can be created (the scheme is shown in Supplementary Figure 3). To correlate the target molecule concentration with drop fluorescence, one of the binding interactions for the activation of the fluorescent reporter should be rate-limiting. These binding interactions can be tuned by using a variety of evolution methods to engineer protein binders with appropriate affinities^14^,^15^.

**Figure 2:**
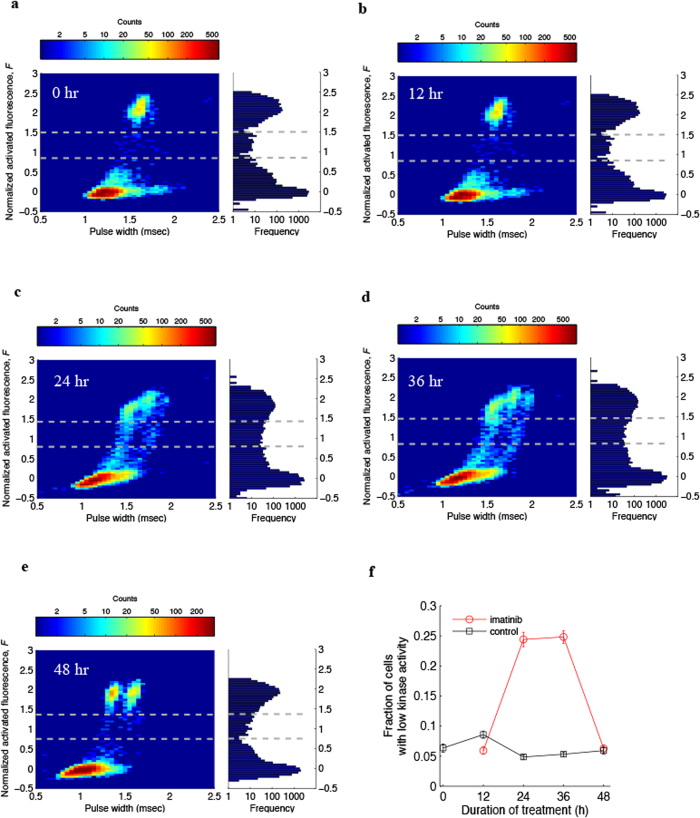
Monitoring kinase activity of drug-treated single leukemia cells using drop-based PAIGE. (**a–e**) Heat maps showing the distribution of drops containing K562 cells treated with imatinib for 0, 12, 24, 36 and 48 hours (left). The corresponding fluorescence histograms are shown on the right. The dash lines separate three groups of drops: bright and low-fluorescence drops containing cells that express high and low kinase activities, respectively, and dark drops without cells. (**f**) Fractions of cells with low kinase activity as determined by drop-based PAIGE during the treatment of imatinib (red circle) or DMSO (black square) as control.

In addition to quantifying proteins, drop-based PAIGE can also be designed to detect enzymatic activity from single cells. To demonstrate this versatility, we customize drop-based PAIGE to detect Abl tyrosine kinase activity present in single chronic myelogenous leukemia cells (K562). Here the interaction between the two fusion proteins is mediated through phosphorylation of a substrate peptide by Abl tyrosine kinase (Supplementary Fig. 4a). In microwell PAIGE, we first show that the GFP expression is activated by Abl kinase, as shown in Supplementary Figure 4b. We then verify for drop-based PAIGE that the drop fluorescence increases with the kinase activity by performing titration experiments with increasing amounts of pure Abl kinase (Supplementary Fig. 4c). Next we co-encapsulate single K562 cells with PAIGE and measure the drop fluorescence after incubation. The fluorescence intensity (F) exhibits a trimodal distribution, which includes bright drops, a small middle population of low-fluorescence drops and dark drops, as shown in Fig. 2a. Bright drops contain single cells with high kinase activity, whereas dark drops do not contain cells due to the Poisson loading. Interestingly, the detection of the low-fluorescence drops suggests a small subpopulation of K562 cells has low kinase activity. Using a threshold of 1.5 > F > 0.8 (see Materials and Methods), we determine that ~6% of K562 cells exhibiting low kinase activity. To support this notion, we treat K562 cells with imatinib, an Abl tyrosine kinase inhibitor, and monitor the kinase activity in single cells for 12, 24, 36 and 48 hours (Fig. 2b–e). We use the same threshold of 1.5 > F > 0.8 to quantify the number of drops in the trimodal distributions at different time points in spite of overlapping peaks (Fig. 2b–e). The number of fluorescent drops (bright drops plus low-fluorescence drops) approximates the number of total encapsulated single K562 cells since dark drops generally do not contain cells. As shown in Fig. 2c–f, unlike the DMSO control, where the fraction of cells with low kinase activity remains ~5%, the corresponding fraction of imatinib-treated cells increases dramatically to ~25% after 24 hours, remains at 25% after 36 hours, and falls to ~6% after 48 hours (more details see Supplementary Table 1). These imatinib-sensitive cells undergo drug-induced apoptosis followed by necrosis, as supported by single-cell staining experiments (Supplementary Fig. 5). At 24-hr imatinib treatment, the similar fractions of cells (25% vs 28%, Fig. 2f and Supplementary Fig. 5) exhibiting low fluorescence activity and undergoing apoptosis suggests that the kinase inhibition by imatinib initiates apoptosis in the same population of cells. At 36-hr imatinib treatment, the fraction of cells with low kinase activity remains at 25% (Fig. 2f), whereas the fraction of apoptotic cells increases to almost 50% (Supplementary Fig. 5). We speculate that at this time point, half of these apoptotic cells have completely lost their kinase activity and cannot be detected by the drop-PAGE assay. At 48-hr imatinib treatment, the fractions of both low-kinase-activity cells and apoptotic cells fall to around ~6% (Fig. 2f and Supplementary Fig. 5). We hypothesize that at this late stage of drug treatment, the majority of imatinib-sensitive cells have undergone extensive necrosis. These cells are lysed before or during centrifugation, and are subsequently removed from intact cells before the single-cell drop and microscopic analyses (Supplementary Fig. 6). Only intact cells are amenable to these single-cell analysis techniques. After 48 hours, the remaining drug-treated cells still exhibit high kinase activity indicating resistance to imatinib (Fig. 2e). These data illustrate that drop-based PAIGE may be used for assessing the responses of heterogeneous cancer cells to drug treatment with single-cell resolution. Furthermore, our drop sorting capabilities allow single-cell genetic analysis of both drug-sensitive and drug-resistant cells^3^.

In summary, we demonstrate a high-throughput mix-and-read label-free method that enables quantification of absolute amounts of proteins in single cells. Current single-cell protein analysis methods measure relative amounts by using antibodies to label/capture target proteins^4^,^7^,^8^,^9^,^16^. Other drop-based single-cell protein analysis methods require co-encapsulating cells with beads coated with capturing antibodies, which complicates the workflow^8^,^9^. Compared to flow cytometry, drop-based PAIGE has the additional advantage of not requiring pretreatment of cells^4^. In contrast to single-cell mass spectrometry methods^4^, our drop-based method preserves cell contents for further genetic analyses, which preserves the link between genotype and phenotype necessary for studies of cell heterogeneity.

## Materials and Methods

### General PAIGE reagents

PAIGE is derived from the *in vitro* two-hybrid system (IVT2H)^11^ and contains 144 nM purified *E.coli* RNA polymerase core enzyme (New England Biolabs), 1.2 μM purified recombinant *E. coli* IHF^17^, 0.8 units/μl murine RNAase inhibitor (New England Biolabs), 0.2 ng/μl (45 pM) plasmid DNA expressing *E. coli* sigma factor 54, 0.2 ng/μl (~40–60 pM) DNA constructs expressing hybrid fusion proteins, 4.4 nM linear reporter DNA expressing the reporter GFP, and the reconstituted protein synthesis system containing T7 RNA polymerase and purified *E. coli* translational components (PURExpress, New England Biolabs)^18^.

### PAIGE for antibody detection

The genes for c-myc epitope peptide (EQKLISEEDL) and a chimeric IgG-binding Fc receptor protein A/G^19^ are commercially synthesized (Integrated DNA Technologies) and cloned into the C-terminus of AD and N-terminus of DB, generating the hybrid fusion proteins, AD-myc and A/G-DB, respectively. As a control, *E. coli* maltose-binding protein (MBP) is also cloned into the C-terminus of AD to give AD-MBP. For antibody detection, DNA constructs expressing AD-myc or AD-MBP and A/G-DB are mixed with PAIGE reagents and different concentrations (2.4–240 nM) of purified monoclonal anti-Myc antibody (Sigma Aldrich) or 240 nM anti-MBP (NEB). For detection in microwell plates (384-well microplate, Corning, Lowell, MA), 10 μl reaction mixtures are incubated at 37 °C for 4 hours and GFP fluorescence (excitation 513 nm, emission 532 nm) is measured using a Spectramax M5 microplate reader (Molecular Devices).

### PAIGE for kinase detection

*In vitro* detection of the protein interaction in response to the phosphorylation of a kinase substrate peptide YEEI by Src kinase has been described previously^11^. In this work, DNA constructs expressing AD-YEEI and SH2-DB are mixed with PAIGE reagents with or without 360 nM purified recombinant Abl kinase (Sigma-Aldrich). To detect the fluorescence, 10 μl of the reaction mixtures are incubated in 384 microwell plates (Corning) at 37 °C for 4 hours and measured using a Spectramax M5 microplate reader (excitation 513 nm, emission 532 nm, Molecular Devices).

### Preparation of cells for single-cell analysis and drug treatments

The hybridoma cell line MYC 1-9E10.2 (9E10) and the leukemia cell line K562 (ATCC) are maintained in Dulbecco’s modified Eagle’s medium DMEM (Cellgro/Mediatech) supplemented with 10% low-endotoxin fetal calf serum (HyClone), 100 U penicillin/ml, and 100 μg/ml streptomycin. Cells are harvested and counted before they are co-flowed into the microfluidic device for single-cell analysis. The workflow for single K562 cell analysis is shown in Supplementary Fig. 6. To inhibit the kinase of K562 cells we incubate them in the presence of 10 μM of imatinib. As a control, K562 cells are cultured in the presence of DMSO. We collect cells at 12, 24, 36, and 48 hours by centrifugation. This centrifugation step separates intact cells, which are collected at the bottom of the centrifuge tube, from lysed cells which remain in the supernatant. We use the Annexin V-FITC Apoptosis Detection Kit (Sigma) to detect apoptosis. This kit also provides propidium iodide to detect necrosis cells as red fluorescence. Cells undergoing apoptosis or necrosis are counted under fluorescence microscopy.

### Fabrication of microfluidic devices

Polydimethylsiloxane (PDMS) microfluidic devices are fabricated using standard soft lithographic methods^20^. The microfluidic channel walls are rendered hydrophobic by treating them with Aquapel (PPG). The electrodes of the PDMS sorting device are made by heating the device to 80 °C and filling the channels with Indalloy 19 (51In, 32.5 Bi, 16.5 Sn; 0.020 inch diameter), a low melting point metal alloy (Indium Corporation)^21^.

### Formation of monodisperse aqueous drops and drop-based PAIGE reactions

We use a microfluidic chip that contains a co-flow drop maker with a cross section of 25um^2^ to generate 35 μm monodisperse aqueous drops in fluorinated oil, HFE-7500 (3 M, *Saint Paul, MN, U.S.A*), containing 1% (w/w) Krytox-PEG diblock co-polymer surfactant (RAN Biotech). The PAIGE reagents and different concentrations (Fig. 1c and Supplementary Fig. 4c) of pure anti-Myc, kinase or cells are encapsulated in drops via co-flow in different channels. To achieve optimal concentrations for PAIGE reactions, we adjust the resistance of the channel for PAIGE reagents to obtain a 4:1 (PAIGE reagents to protein or cells) co-flow ratio. For both pure anti-Myc and cell lysis, we add 25% NP40 to the PAIGE reagents to achieve a 1% working concentration. Off-chip incubation is conducted at 37 °C for 6 hours.

### Fluorescence-activated drop sorting (FADS) analysis

We use a microfluidic fluorescence-activated drop sorter to isolate bright drops (17). After incubation, drops are re-injected into the sorter at a flow rate of 30 μL/h and evenly spaced by HFE-7500 oil with surfactant flowing at a rate of 180 μL/h through a 40 μm x 40 μm channel. When a drop passes through the laser’s focal point, its fluorescence is collected by a microscope objective and focused onto a photomultiplier tube (PMT) (Hammamatsu). The pulse height is used as the measure of drop fluorescence. The pulse width, which is the duration of time for a drop to pass through the laser detector, is used as the measure of drop size. The PMT is connected to a custom computer LabView program running on a real-time field-programmable gate array card (National Instruments). All drops are gated based on pulse width to exclude merged drops. Pulse width is routinely used to plot the data in drop-based microfluidics. Pulse width is proportional to drop size, reflecting the monodispersity of drops. Encapsulation of cells or other materials can affect the local hydrodynamics of the pinch-off process, resulting in changes of the drop size, and thus the observed pulse width. To protect the sorted drops from evaporation and facilitate their handling, we collect them into a tip loaded with 20 μl of drops containing pure water. We then isolate single sorted drops by distributing the emulsion into microwells whose number is determined by Poisson loading. For example, we distribute 10 bright drops into 30 microwells.

For K562 cells after drop-based PAIGE reactions, drops exhibit a trimodal distribution in fluorescence (Fig. 2a). To quantify the number of bright drops, low-fluorescence drops and dark drops (Supplementary Table 1), we use the distribution without drug treatment to define the threshold for each population of drops, since it has relatively well-defined and separate peaks (Fig. 2a). We then apply the same threshold to the distributions at 12, 36 and 48 hours. Specifically, we set the fluorescence threshold at the first minimum (F = 0.8) of the trimodal distribution to distinguish between low-fluorescence drops and dark drops, and at the second minimum (F = 1.5) of the trimodal distribution to distinguish between low-fluorescence drops and bright drops.

### RT-PCR amplification of mRNA from single sorted hybridoma 9E10 cells and Sanger sequencing

We break the emulsion in the microwells with 10 μL of 20% of 1H,1H,2H,2H-perfluorooctanol (Alfa Aesar) followed by vortexing and centrifugation. Five microliters of ddH_2_O is added to each well. We then pipette out the aqueous phase containing cellular RNA for RT-PCR. The 25 μL RT-PCR cocktail is composed of 1 μL of OneStep RT-PCR Enzyme Mix with 1× buffer (Qiagen), 400 μM dNTPs, 0.25 μM β2-macroglobulin forward and reverse primers, and 5 μL of the cellular RNA. The RT-PCR is done in one step by combining RT and PCR reactions. Thermocycling conditions are: 50 °C for 30 min, 95 °C for 10 min, 40 cycles of 95 °C for 30 s, 58 °C for 30 s, and 72 °C for 40 s, followed by 72 °C for 5 min. The PCR products are purified with GenElute™ Gel Extraction Kit (Sigma) and sent out for Sanger sequencing.

## Additional Information

**How to cite this article**: Abbaspourrad, A. *et al.* Label-free single-cell protein quantification using a drop-based mix-and-read system. *Sci. Rep.* **5**, 12756; doi: 10.1038/srep12756 (2015).

## Supplementary Material

Supplementary Information
