# Supplementary material for: Label-free single-cell protein quantification using a drop-based mix-and-read system
Source: Sci Rep. 2015 Aug 3;5:12756. doi: 10.1038/srep12756 (PMC4522677; doi:10.1038/srep12756)
Supplement: Supplementary Information [file srep12756-s1.pdf]

Supplementary Information

Label-free single-cell protein quantification using a drop-based mix-and-read system

Alireza Abbaspourrad[1,6], Huidan Zhang[1,2,6], Ye Tao[1,3,6], Naiwen Cui[1], Haruichi Asahara[5], Ying Zhou[5], Dongxian Yue[5], Stephan Koehler[1,4], Lloyd Ung[1], John Heyman[1], Yukun Ren[3], Roy Ziblat[1], Shaorong Chong[5] and David A. Weitz[1,4]

Supplementary Fig. 1 (Chong)

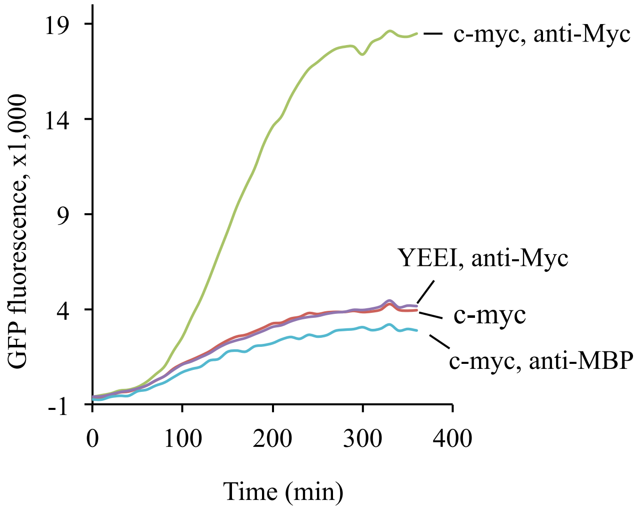

Supplementary Fig. 1. Detection of anti-Myc in a microwell plate using PAIGE. The GFP fluorescence of PAIGE reactions is monitored during incubation at 37°C in a microplate reader. Reactions expressing the c-myc epitope are performed in the presence or absence of 240 nM anti-Myc. As additional negative controls, we also monitor the fluorescence of reactions expressing c-myc in the presence of 240 nM anti-MBP, and YEEI peptide in the presence of 240 nM anti-Myc.

Supplementary Fig. 2 (Chong)

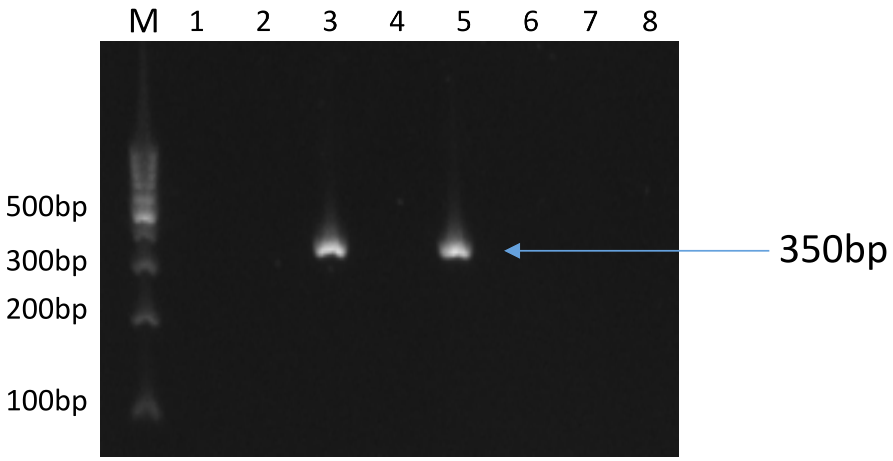

Supplementary Fig. 2. Agarose gel image of amplification products from RT-PCR of a 350 bp region of mRNA encoding β2-macroglobulin from single cells. M: 100 bp DNA marker. We sort 3 bright drops into 10 μl water drops, and distribute them into 8 wells for RT-PCR (lanes 1 to 8). We successfully amplify the products from two bright drops and verify their sequences by Sanger sequencing. The third drop is possibly lost during drop manipulation.

Supplementary Fig. 3 (Chong)

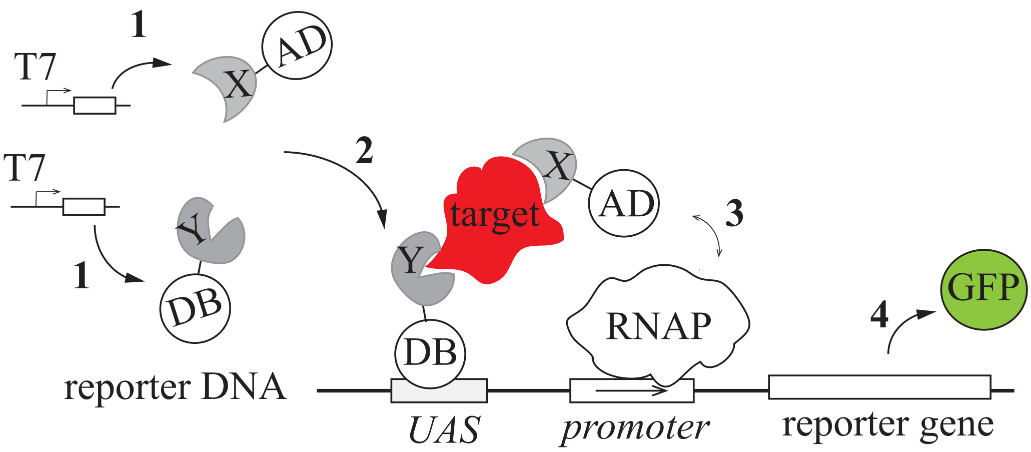

Supplementary Fig. 3. Scheme of universal PAIGE for detecting and quantifying any target molecule of interest in solution. Appropriate binders (X and Y) in the fusion proteins are expressed from DNA templates (step 1) and bind to different regions of a target (red). Such interactions result in a ternary complex that binds to the upstream-activation sequence (UAS) on the reporter DNA and recruits AD near the promoter-bound RNA polymerase (RNAP) (step 2). AD activates RNAP (step 3) to express the reporter gene, which produces GFP (step 4). If one of the binding interactions (X/target or Y/target) is rate-limiting for the reactions, the concentration of the target should be linearly correlated to the GFP concentration.

Supplementary Fig. 4 (Chong)

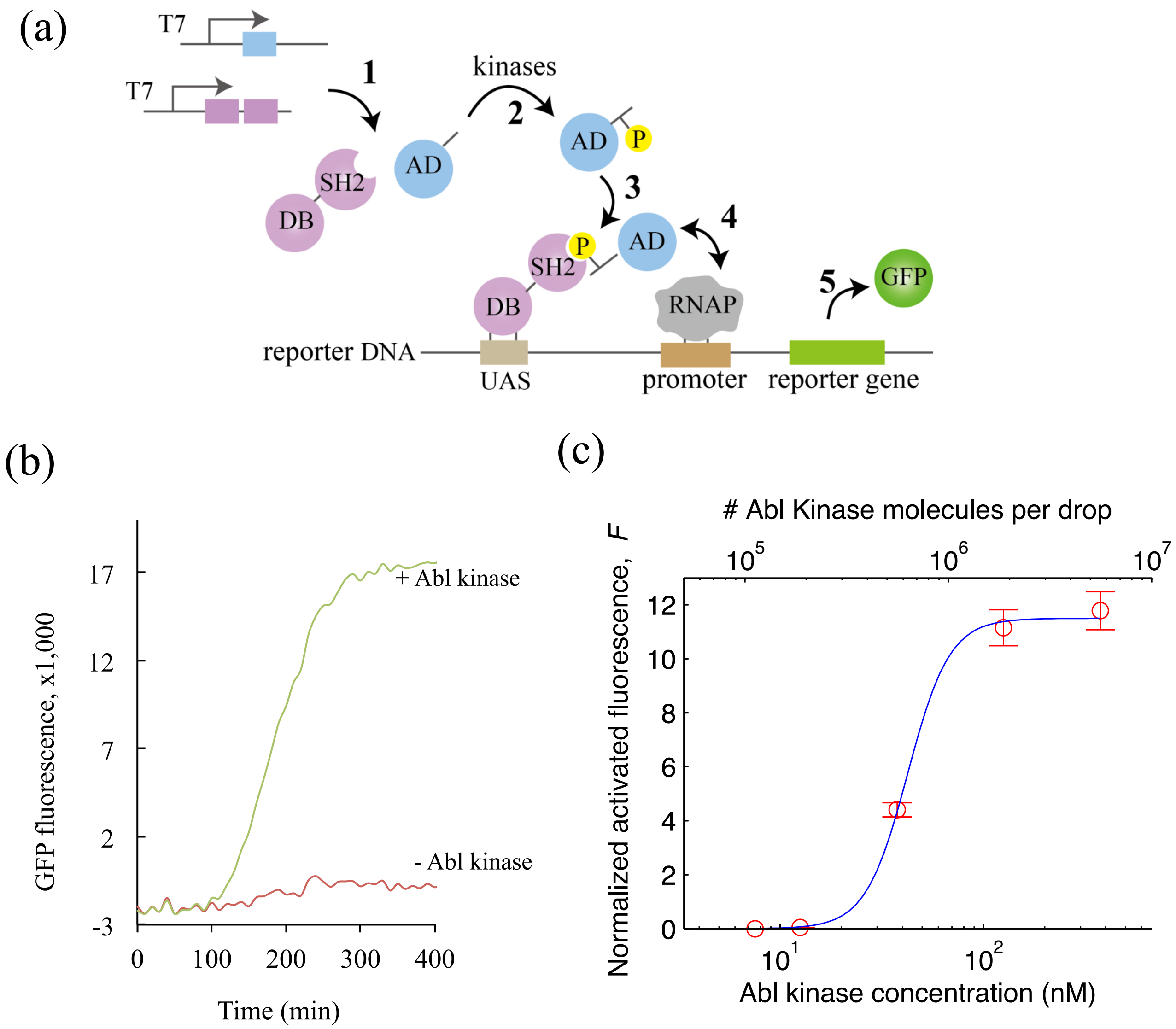

Supplementary Fig. 4. (a) Scheme of PAIGE for detecting kinase activity. AD is fused to a kinase substrate peptide and SH2 is fused to DB. Both fusion proteins, AD-peptide and SH2-DB, are constitutively expressed under T7 promoters from input DNA templates (step 1). Phosphorylation of the substrate peptide by a kinase (step 2) results in the peptide's binding to SH2-DB which is bound to the upstream-activation sequence (UAS) on the reporter DNA (step 3). AD activates the promoter-bound RNA polymerase (RNAP) (step 4) to express the reporter gene, which produces GFP (step 5). **(b)** Detection of Abl kinase activity in a microwell plate using PAIGE. The GFP fluorescence is monitored during incubation at 37°C in a microplate reader. **(c)** Titration curve of pure Abl kinase using drop-based PAIGE. The fluorescence of drops with Abl kinase is normalized by that of drops without Abl kinase. The normalized activated fluorescence, *F*, is obtained by subtracting one.

Supplementary Fig. 5 (Chong)

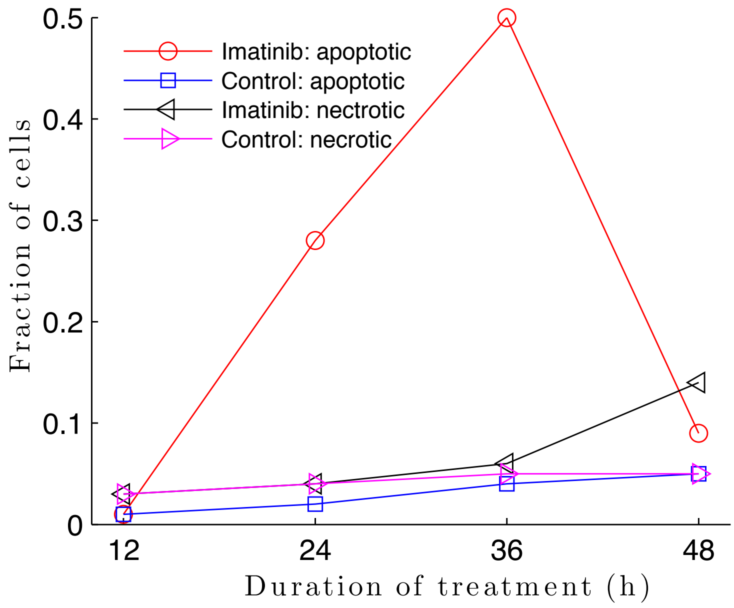

Supplementary Fig. 5. Fractions of leukemia K562 cells undergoing apoptosis and necrosis during drug treatments as determined by cell staining. The cells are treated with 10 μM imatinib and DMSO is the control. The fraction of apoptotic cells increased steadily during first 36 hours due to kinase inhibition of imatinib. After 36 hours, the apoptotic fraction drops dramatically because in this time interval the the imatinib-sensitive cells become necrotic and are removed by centrifugation during the sample preparation process.

Supplementary Fig. 6 (Chong)

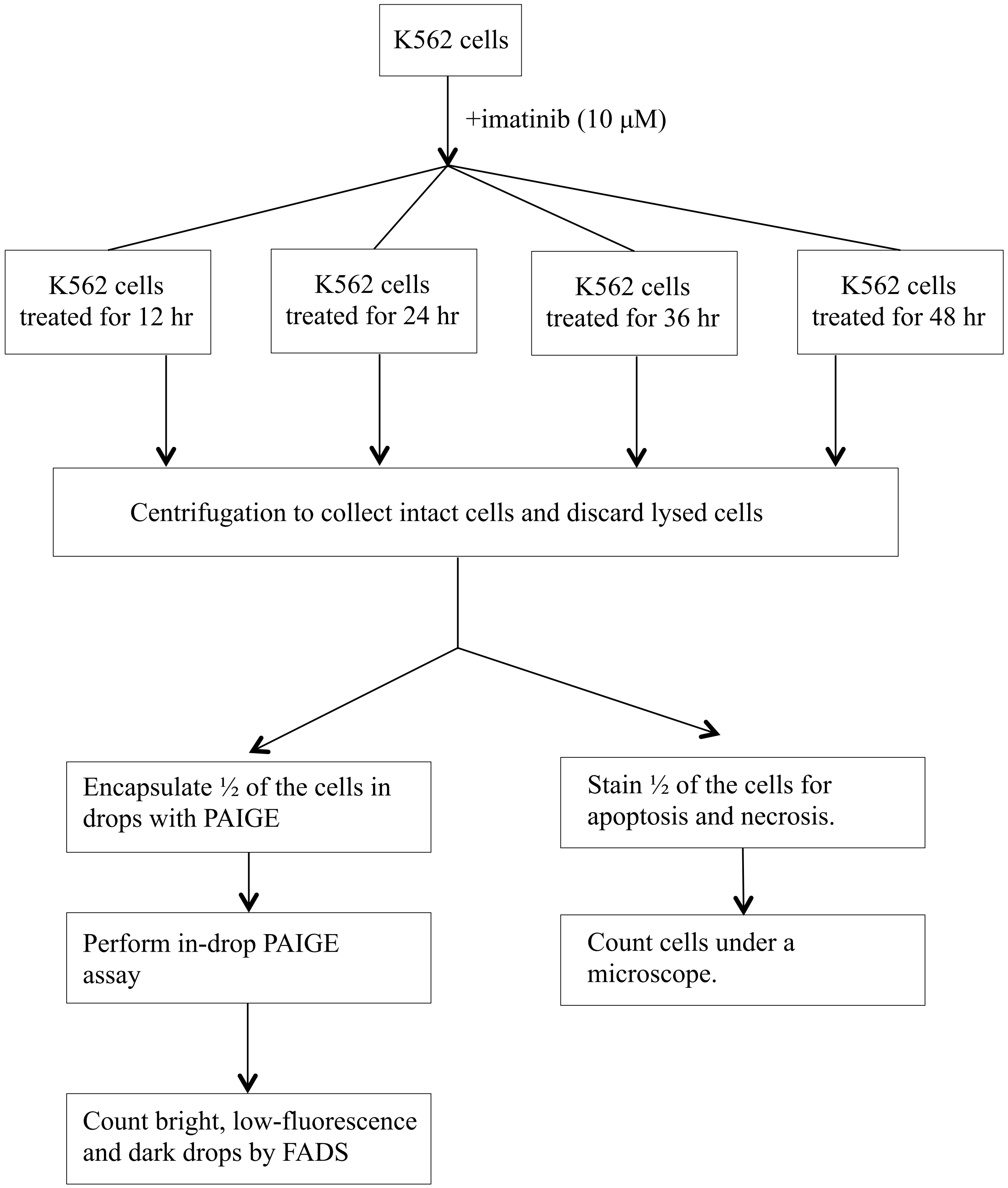

Supplementary Fig. 6. Work flow of the experimental steps for monitoring the kinase activity, apoptosis and necrosis of single K562 cells during imatinib treatment.

Supplementary Table 1. The numbers of drops counted by drop-based PAIGE

A. Cells treated with imatinib

imatinib (10 μM)	0 hr	12 hr	24 hr	36 hr	48 hr
Low-fluorescence drops	90	92	409	549	106
Bright drops	1458	1460	1267	1663	1583
Total fluorescent drops (excluding dark drops)	1548	1552	1676	2212	1689
Fraction of low-fluorescence drops in total fluorescent drops	5.8%	5.9%	24.5%	24.8%	6.3%

B. Control cells without drug treatment

	0 hr	12 hr	24 hr	36 hr	48 hr
Low-fluorescence drops	89	212	190	230	92
Bright drops	1312	2259	3722	4097	1460
Total fluorescent drops (excluding dark drops)	1401	2471	3912	4327	1554
Fraction of low-fluorescence drops in total fluorescent drops	6.4%	8.6%	4.9%	5.3%	5.9%
